# Metabolomic Profile of *Arthrospira platensis* from a Bulgarian Bioreactor—A Potential Opportunity for Inclusion in Dietary Supplements

**DOI:** 10.3390/life14020174

**Published:** 2024-01-24

**Authors:** Krastena Nikolova, Galia Gentscheva, Desislava Gyurova, Vera Pavlova, Ivayla Dincheva, Margarita Velikova, Anelia Gerasimova, Lubomir Makedonski, Georgi Gergov

**Affiliations:** 1 Department of Physics and Biophysics, Medical University—Varna, 9000 Varna, Bulgaria; 2 Department of Chemistry and Biochemistry, Medical University—Pleven, 5800 Pleven, Bulgaria; 3 Institute of General and Inorganic Chemistry, Bulgarian Academy of Sciences, 1113 Sofia, Bulgaria; 4 Department of Analytical and Laboratory Activities, National Center of Public Health and Analyses—Sofia, 1431 Sofia, Bulgaria; d.giurova@ncpha.government.bg (D.G.);; 5 Department of Agrobiotechnologies, Agrobioinstitute, Agricultural Academy, 1164 Sofia, Bulgaria; ivadincheva@yahoo.com; 6 Department of Physiology, Medical University—Varna, 9000 Varna, Bulgaria; msvelikova@yahoo.com; 7 Department of Chemistry, Medical University—Varna, 9000 Varna, Bulgaria; anelia.gerasimova@mu-varna.bg (A.G.); lubomir60@yahoo.com (L.M.); 8 Institute of Chemical Engineering, Bulgarian Academy of Sciences, Acad. Georgi Bontchev Str., Bl.103, 1113 Sofia, Bulgaria; ggergov187@gmail.com

**Keywords:** *Arthrospira platensis*, vitamins, amino acids, fatty acids, sterol content, elemental composition

## Abstract

The present study aims to elucidate the metabolomic profile of *Arthrospira platensis* grown in a bioreactor in Bulgaria. The results show that *Arthrospira platensis* has a high content of mannose, 137.02 mg g^−1^, and vitamin A (retinol)—10.3 μg/100 g. High concentrations of calcium, sulfur, and zinc distinguish its elemental composition. The freeze-dried powder contained 15.81 ± 0.45% dietary fiber, 50.16 ± 0.25% total protein content, and 1.22 ± 0.11% total fat content. Among the unsaturated fatty acids with the highest content is α-linolenic acid (25.28%), while among the saturated fatty acids, palmitic acid prevails (22.55%). Of the sterols in the sample, β-sitosterol predominated. There is no presence of microcystins LR, RR, YR, and nodularin. Therefore, *Arthrospira platensis* grown in a Bulgarian bioreactor is safe for use in the pharmaceutical and food industries. Many of the organic compounds found have applications in medicine and pharmacology and play an important role in biochemical processes in the body. Therefore, *Arthrospira platensis* grown in Bulgaria has a high potential for use as an independent food supplement or in combination with other natural products.

## 1. Introduction

A lack of micronutrients can lead to irreversible health consequences. Vitamin deficiencies in women of reproductive age, infants, and children require therapeutic doses, which can be obtained through various nutritional supplements [1]. Food supplements supplement the diet with vitamins, fiber, fatty acids, amino acids, etc. Supplementation with a higher protein content may reduce the length of time spent in hospital, rehabilitation, and the number of complications [2].

Microalgae and cyanobacteria contain a wide range of amino acids, fatty acids, minerals, vitamins, fibers, flavonoids, and sugars [3,4,5]. This wide range of nutrients makes them suitable for inclusion in food products or dietary supplements used in sustainable dietary regimes. In addition to a series of positive effects on human health, they contribute to combating the overweight problem affecting a significant portion of the world’s population, improving environmental cleanliness, and aiding in the development of developing areas with bioreactors.

One of the most common types of cyanobacteria is *Arthrospira platensis* (commercially known as *Spirulina*), which can be found in both freshwater and saltwater environments [6]. The study of its composition is relevant as it depends on environmental conditions, light intensity, and more. *Arthrospira platensis* finds applications in the development of dietary supplements and pharmaceutical and cosmetic products [7]. *Arthrospira platensis* is added to foods due to its foaming, gelling, and emulsifying properties [8]. Some of the literature suggests that *Arthrospira platensis* reduces blood sugar [9] and LDL cholesterol levels in the body [10]. It also causes a clear improvement in metabolic processes, contributing to weight reduction [10]. In recent years, research has indicated that *Arthrospira platensis* can be successfully used in the development of probiotics [11] and dietary supplements that have a positive impact on mental and physical fatigue [12] while protecting living organisms from the harmful effects of heavy metals [13]. Some reviews report that the consumption of *Spirulina* in nutritional supplements accelerates muscle recovery from exercise fatigue in athletes, protects them from exercise-induced oxidative stress, and stimulates the immune system [14]. There is also evidence of antioxidant, immunomodulatory, and anti-inflammatory effects when *Spirulina* supplements are included in patients’ diets [15].

The present study aims to create a metabolomic profile of *Arthrospira platensis* grown in a bioreactor in Bulgaria and to evaluate the possibility of its use in the development of dietary supplements.

## 2. Materials and Methods

### 2.1. Materials Used

The *Arthrospira platensis*, cultivated in a bioreactor in Bulgaria (near Varvara village), was studied after convective drying. The conditions for growth and the habitat of *Arthrospira platensis* were described by G. Gentscheva et al. in the following steps: creating sowing, changing the habitat of the samples from the laboratory into the production conditions, and growing the samples into a large volume [16]. In the processing workshop, a centrifuge separated them from the water. After that, they were dried in a thin layer with transversely oriented airflow towards the product layer at 45 ± 2 °C and relative humidity of the circulating air, on average 10%. The samples reaching a constant mass indicates the end of the drying process.

**Figure 1:**
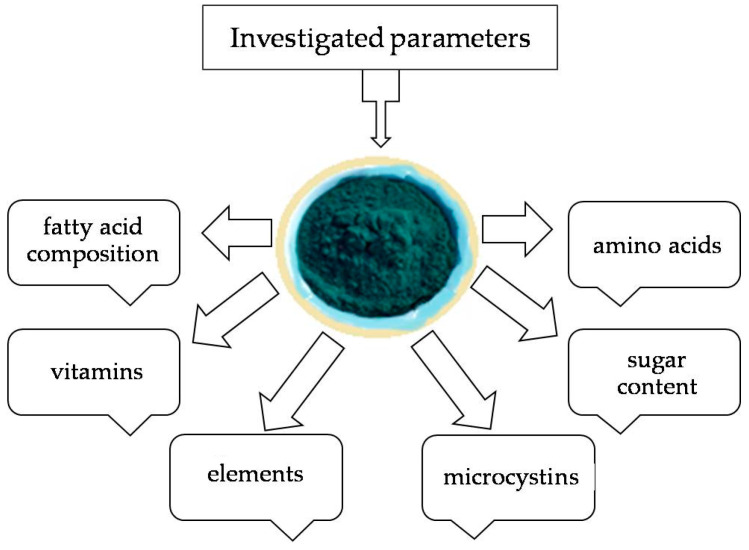
Summary of investigated parameters of *Arthrospira platensis* from Bulgarian bioreactor.

Investigated parameters are shown in Figure 1.

### 2.2. Sample Preparation

**Figure 2:**
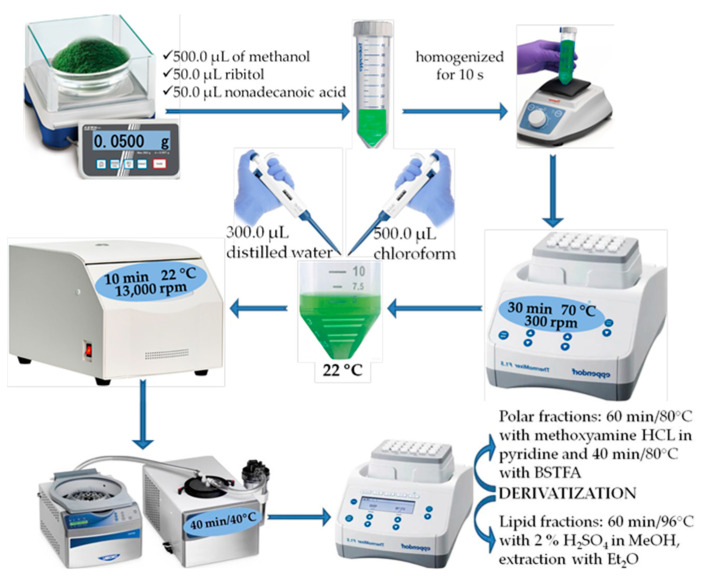
Sample handling process.

In total, 50.0 mg of *Spirulina* was weighed, and 500.0 µL of methanol, along with 50.0 µL of the internal standards ribitol and nonadecanoic acid, was added at a 1.0 mg mL^−1^ concentration. The mixture was homogenized with a Vortex (IKA^®^ VORTEX 3, Darmstadt, Germany) for 10 s, followed by incubation for 30 min at 70 °C and 300 rpm on the centrifuge. The mixture was then cooled to room temperature, and 300.0 µL of distilled water and 500.0 µL of chloroform were added. The sample was centrifuged for 10 min at 22 °C and 13,000 rpm (Figure 2).

#### 2.2.1. Polar Phase

In total, 300.0 µL of the supernatant was pipetted and evaporated under vacuum until dry. Then, 100.0 µL of a methoxyamine solution (20 mg mL^−1^) was added to the residue. Incubation was carried out under the following conditions: 60 min at 70 °C with stirring at 300 rpm. Then, 50.0 µL of the silylation reagent BSTFA was added. The mixture was incubated again at 70 °C for 40 min, then cooled to room temperature and mixed with 300 µL of chloroform. Finally, a 1.0 µL volume from the resulting solution was withdrawn and introduced into the chromatographic system.

#### 2.2.2. Nonpolar Phase

A total of 300.0 µL was pipetted and evaporated under vacuum to dryness; then, 1.0 mL of a solution of sulfuric acid in 2% methanol was added. The mixture was incubated under the following conditions: 60 min at 90 °C with stirring at 300 rpm. After cooling to room temperature, a triple extraction with hexane (3 × 300 µL) was performed. Then, the samples were dried under vacuum at 30 °C. Next, 50.0 µL of pyridine and 50.0 µL of the silylation reagent BSTFA were added. The mixture was incubated again at 70 °C for 40 min, cooled to room temperature, and mixed with 300 µL of chloroform. Finally, a 1.0 µL volume from the solution was withdrawn and introduced into the chromatographic system.

### 2.3. Methods Used

#### 2.3.1. Microcystin Analysis

The dry biomass sample was prepared for extraction by weighing approximately 20 mg of *Arthrospira platensis* into a 2 mL plastic bottle with a cap (Eppendorf type). Then, 1.5 mL of 50% methanol (Lichrosolv, HPLC gradient grade, Merck KGaA, Darmstadt, Germany) was added to the sample, along with ultrapure water with a conductivity of 0.055 µS (*v*/*v*). The sample was subjected to ultrasonic extraction in a bath for 1 h, followed by centrifugation. The resulting extract (0.5 mL) was filtered using PTFE filters (0.45 µm, Thermo Fisher Scientific Inc., Waltham, MA, USA) prior to chromatography. Two parallel extractions were prepared.

Chromatographic determination was performed using an Agilent 1200 liquid chromatograph equipped with a diode array detector. The analytical column used was a Supelcosil ABZ + Plus column (150 mm × 4.6 mm, 5 µm, Supelco, Darmstadt, Germany), thermostated at 25 °C. A gradient of acetonitrile (Lichrosolv, HPLC gradient grade, Merck KGaA, Darmstadt, Germany) and ultrapure water (conductivity of 0.055 µS) was employed, with the addition of 0.1% trifluoroacetic acid (Trifluoroacetic acid, suitable for HPLC ≥ 99.0%, Merck KGaA, Darmstadt, Germany) to both solvents. The gradient profile included 20% acetonitrile at 0 min, which increased to 46% at 20 min, with a total chromatography time of 25 min at a flow rate of 1 mL min^−1^. Detection was performed at a wavelength of 238 nm.

#### 2.3.2. Gas Chromatography and Mass Spectrometry

The solutions obtained during the sample preparation in Section 2.2.1 and Section 2.2.2 were injected into a system consisting of a gas chromatograph 7890A (Agilent Technologies, Santa Clara, CA, USA) and a mass spectrometric detector 5975C (Agilent Technologies). The column used was HP-5 ms with the following parameters: length 30 m, diameter 0.32 mm, and a film thickness of 0.25 µm. The following temperature program was used: initial temperature 60 °C, no hold time, ramp to 300 °C at 5 °C min^−1^, and hold for 10 min. The injector and detector temperatures were set at 250 °C; helium was used as the carrier gas with a flow rate of 1.0 mL min^−1^; the mass detector scanning range was *m*/*z* = 50–550; a 1 µL sample volume was injected in split mode (split 1:10).

The compounds were identified by comparing retention times and Kovats indices with those of standard substances and mass spectrometric data from the NIST’08 library (National Institute of Standards and Technology, Gaithersburg, MD, USA). The concentrations were determined using the internal standard method in µg g^−1^ dry weight.

#### 2.3.3. Determination of Vitamin Content and Protein

##### Determination of Proteins

Protein determination was conducted using an in-house test method LMI 01.11 (Kjeldahl method). The sample was mineralized, which involved weighing approximately 1 g of the sample into a Kjeldahl flask and adding concentrated sulfuric acid and a catalyst tablet in a Kjeldahl apparatus. The mineralization process takes about 2 h at a temperature of 419 °C, followed by cooling of the sample and neutralization with 40% sodium hydroxide. Ammonia was absorbed in a 4% boric acid solution, followed by titration with 0.1 N hydrochloric acid. The obtained total nitrogen content was multiplied by a conversion factor of 6.25 and expressed in % or g/100 g.

##### Determination of Vitamin B_2_

The determination of riboflavin (vitamin B_2_) was performed after acid hydrolysis to release vitamin B_2_ and subsequent high-performance liquid chromatography (HPLC) with fluorometric detection. The hydrolysis was conducted by weighing approximately 2 g of the sample on an analytical balance with an accuracy of 0.0001 g and dissolving it in a 20 mL hydrochloric acid (HCl) solution (0.1 mol L^−1^). Hydrolysis was carried out in a water bath at a temperature of 95–100 °C for 40 min. After hydrolysis, the sample was centrifuged at 1200 rpm, filtered with a 0.45 micron filter, and then subjected to HPLC (Perkin Elmer series 200 with Zodiac C18 column (5 µm, 4.6 × 250 mm)). The conditions under which liquid chromatography was conducted were as follows: working temperature 20 °C; eluents: methanol for HPLC; phosphate buffer (25 mM), solution pH = 2÷3 (2.5), in a ratio of 50:50 (*v*/*v*); flow rate 0.8 mL min^−1^; detection with fluorimetric detector, type 3000 FL, λx = 453 nm; and λm = 520 nm. Vitamin B_2_ was identified by comparing retention times and quantitatively determined in mg/100 g relative to a reference solution of riboflavin (vitamin B_2_) with a known concentration. 

##### Determination of Vitamin E and Vitamin A

The content of vitamin E (α-tocopherol), mg/100 g, was determined according to the standard [17].

On an analytical balance, approximately 5 g of the sample was weighed into a round-bottom flask with an accuracy of 0.0001 g. Absolute ethanol, antioxidant BHT, and a potassium hydroxide solution were added. Saponification was carried out by heating in a water bath at a temperature of 95–97 °C for 45 min, under a reflux condenser and in a nitrogen atmosphere. After cooling, the sample underwent triple extraction with diethyl ether, washing of the extract with ultrapure water, and passing through anhydrous sodium sulfate. The extract was evaporated to dryness using a vacuum rotary evaporator at 40 °C, dissolved in methanol for HPLC analysis to a specific volume, and filtered through a 0.45 μm filter before chromatography.

Liquid chromatography was performed using a high-pressure liquid chromatograph: Perkin Elmer, series 200, with a Zodiac C18 column (5 µm, 4.6 × 250 mm). The conditions for determining the content of vitamin E were as follows: working temperature 20 °C; eluent: methanol for HPLC, 100%; flow rate 0.6 mL min^−1^; and detection with fluorescence detector type 3000 FL, with excitation wavelength (λex) of 285 nm and emission wavelength (λem) of 345 nm. Vitamin E (α-tocopherol) in the sample was identified by comparing the retention time and quantitatively determined in mg/100 g compared to a reference solution of vitamin E (α-tocopherol) with a known concentration. When determining the content of vitamin A, there were differences in the conditions for conducting liquid chromatography, namely, a working temperature of 20 °C; eluent: methanol for HPLC and ultrapure water, in a ratio of 98:2 (*v*/*v*); flow rate 0.6 mL min^−1^; detection with a fluorimetric detector type 3000 FL; and wavelength λex = 325 nm and λem = 475 nm. Vitamin A (as retinol) in the sample was identified by comparing the retention time and quantitatively determined in µg/100 g compared to a reference solution of retinol (vitamin A) ≥ 95.0% with a known concentration.

##### Determination of Vitamin C

The determination of ascorbic acid (vitamin C) was carried out after extraction with meta-phosphoric acid, aiming to release vitamin C, followed by HPLC with UV detection. For this purpose, a sample weighing between 1 and 2.5 g was accurately weighed on an analytical balance. It was then filled up to 2/3 of the flask volume with 3% meta-phosphoric acid, followed by extraction in an ultrasonic bath for 20 min. The solution was adjusted to a specific volume with meta-phosphoric acid, and then it was centrifuged using an ultracentrifuge at 1200 rpm. Before chromatography, the solution was filtered through a 0.45 μm filter. The chromatographic conditions involved using a high-pressure liquid chromatograph Perkin Elmer series 200; chromatographic column C19305; operating temperature of 20 °C; eluent: phosphate buffer: 3 M KH_2_PO_4_ in 0.35% orthophosphoric acid; flow rate of 0.5 mL/min; and detection with UV detector at a wavelength of 248 nm. Vitamin C in the sample was identified by comparing the retention time and quantitatively determined in mg/100 g compared to a reference solution of ascorbic acid (vitamin C) with a known concentration.

#### 2.3.4. Determination of Fat in *Arthrospira platensis*

##### Preliminary Acid Hydrolysis

Between 5 and 10 g of the sample was accurately weighed to 0.0001 g in a round-bottom flask. The weighed sample was added to 50 mL of hydrochloric acid, and a few grains of pumice stone were added to ensure even boiling in the flask. The flask was equipped with a reflux condenser and immersed in a boiling water bath, heated to boiling for 15 min. After the reaction time elapsed, the reflux condenser was rinsed with warm distilled water, and the flask was removed. The solution was filtered through a previously wetted filter with distilled water. The filter and its contents were washed with warm distilled water until a negative reaction for chloride ions (qualitative reaction with a silver nitrate solution) was achieved. The washed filter, along with its contents, was dried in an oven at (105 ± 5) °C for 1 h. The dried filter with its contents was carefully folded and transferred to an extraction thimble for subsequent Soxhlet extraction.

##### Soxhlet Extraction, after Acid Hydrolysis

The thimble with the dried sample was placed in a Soxhlet extractor. The required amount of petroleum ether was added for the proper extraction, which takes about 3–4 h. After the extraction was completed, the flask was connected to a vacuum rotary evaporator to remove the solvent. The flask, with the isolated fat at the bottom, was dried in an oven at (105 ± 5) °C for 1 h, then cooled and weighed. The mass of the separated fat was calculated in g/100 g.

#### 2.3.5. Determination of Fatty Acid Composition in *Arthrospira platensis*

Fatty acid composition was determined according to standards [18,19].

##### Fat Extraction

Approximately 10 g of the sample was mixed with 50 mL of methanol and 40 mL of chloroform, with no additional water added. The sample was left to stand at room temperature for 24 h. On the following day, 20–30 mL of an organic solvent (chloroform) was added to the sample. The sample was extracted with vigorous stirring for 5 min and then filtered through a Büchner funnel. The filter was re-extracted with the appropriate organic solvent; then, the extract was filtered, with washing, and the lipid extracts were combined. The aqueous methanol layer and the organic solvent layer with the lipids were separated, either by centrifugation or gravimetrically. The lipid solution was filtered through anhydrous sodium sulfate, with further washing using the organic solvent, ensuring that the final volume of the lipid solution did not exceed 100 mL. A 1 mL portion of the lipid solution and the solvent was taken and evaporated under nitrogen.

##### Preparation of Methyl Esters of Fatty Acids with 20% Boron Trifluoride in Methanol

The sample prepared for methylation was mixed with 5 mL of 0.5 N sodium hydroxide methanol solution, and the sample was saponified for about 10 min under reflux in a water-cooled condenser. When the sample was completely saponified, the solution remained clear. After saponification, the sample was cooled to 75 °C, and 6 mL of a 20% boron trifluoride solution in methanol was added through a condenser. The methylation process was carried out for 5 to 10 min at a temperature of 70–75 °C. Upon completion of methylation, the flask was cooled, and 4–5 mL of n-hexane and 20 mL of distilled water were added. The methyl esters of the fatty acids were extracted twice with n-hexane, and the aqueous methanol layer was separated from the n-hexane layer. The n-hexane layers were combined, dried, and neutralized by adding anhydrous sodium sulfate and sodium carbonate.

##### Gas Chromatographic Determination of the Fatty Acid Composition

A 1 µL portion of the analyzed sample was injected into a gas chromatograph. Each of the resulting peaks in the chromatogram was identified, and its content was calculated relative to standards of methyl esters of fatty acids with compositions.

#### 2.3.6. Determination of Dietary Fiber According to AOAC 985.29 Method

To two parallel samples of dried *Arthrospira platensis*, each weighing approximately 1 g (if they contained more than 10% fat, they were extracted with petroleum ether), 40 mL of 0.08 M phosphate buffer was added. The samples were gelatinized with thermostable α-amylase and enzymatically digested with protease and amyloglucosidase to remove protein and starch. The samples were transferred to preheated and preweighed crucibles. The residue on the crucibles was washed with 78% and 95% ethyl alcohol and acetone, respectively. The crucibles were placed in an oven and dried overnight at 105 °C, after which the residue was weighed. One duplicate (crucible) was analyzed for residual protein, and the other was ashed for 5 h at 525 °C for ash content. In parallel, blank samples were prepared, containing the same reagents, with phosphate buffer used instead of food. After corrections for protein and ash, the dietary fiber content was expressed in g/100 g.

#### 2.3.7. Elemental Composition

A sample of about 0.3 g was weighed for analysis balance in Teflon containers for the microwave digestion system. In total, 10 mL of 65% HNO_3_ (Suprapur^®^, Merck, Darmstadt, Germany) was added. Microwave digestion was carried out according to the following procedure: 180 °C was reached in 10 min and maintained for 10 min. After cooling, the solution was transferred to a 25 mL volumetric flask and diluted to the mark with deionized water. A blank sample also went through the entire analytical procedure [20].

The content of Mg, Fe, Mn, Cu, Ba, Ca, Cr, Sr, S, and Zn was measured by the ICP-OES system ULTIMA 2, (Jobin Yvon, Longjumeau, France). The multi-element standard solution IV for ICP (*Trace*CERT^®^, Merck, Darmstadt, Germany) and S − 1 g L^−1^ for ICP (*Trace*CERT^®^, Merck, Germany) were used to prepare diluted working standard solutions for instrument calibration.

## 3. Results and Discussion

The sample was analyzed for the presence of microcystins LR, RR, YR, and nodularin. The analysis results do not show the presence of the specified toxins at the detection limit of 0.2 µg L^−1^. Therefore, *Arthrospira platensis* grown in a Bulgarian bioreactor is safe for use in the pharmaceutical and food industries.

The lyophilized powder of *Arthrospira platensis* contained 15.81 ± 0.45% dietary fiber, 50.16 ± 0.25% total protein, and 1.22 ± 0.11% total fat.

**Scheme 1:**
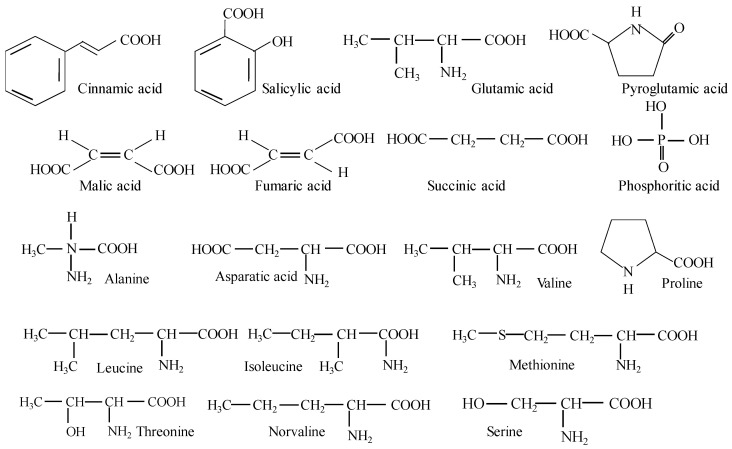
Phenolic acids, carboxylic acids, and non-essential and essential amino acids.

The main classes of chemical compounds in *Arthrospira platensis* are presented in Table 1. Structural formulas of phenolic acids, carboxylic acids, and non-essential and essential amino acids are shown in Scheme 1.

*Arthrospira platensis* contains over 50% easily digestible proteins with high biological value [23], including essential amino acids such as leucine, isoleucine, valine, tryptophan, and methionine [24,25,26,27]. It has been shown that the cyanobacterium grown in a Bulgarian bioreactor contains 1.5 to 2 times higher levels of essential amino acids compared to those reported [22]. 

Phenolic compounds in *Arthrospira platensis* are present in lower concentrations compared to those in brown and red algae [28]. This fact is confirmed in our study, where phenolic acids were found at concentrations of around 3%. Among them, cinnamic acid is the most abundant (approximately 2.3%). This compound can be utilized in creating drugs with antioxidant and anti-inflammatory properties. Aspartic acid, the non-essential amino acid found in the highest concentration (approximately 5.25%), can serve as a component for regulating the pH of pharmaceutical products.

Besides phenolic compounds, which are crucial for the antioxidant activity of the product, knowledge about the contained vitamins is of great significance as they act as precursors to enzymatic cofactors that support the body’s metabolic functions. Table 2 summarizes the data on the vitamin content in the examined sample of *Arthrospira platensis*.

The content of vitamin E is comparable and sometimes higher than that in terrestrial plants [29]. The literature indicates a range of 5–20 mg/100 g [30]. In our case, it is 3.57 mg/100 g. This vitamin acts as an antioxidant, protecting membrane lipids from oxidation and damage [31]. It plays a chemoprotective role and inhibits tumor growth [32], including the development of prostate tumor cells [32]. It also enhances endocrine function and strengthens the epithelial tissue of blood vessels [33].

Vitamin A is associated with an anti-inflammatory effect and has a protective effect on the development of prostate cancer [34]. An additional 2 mg per day of provitamin A (β-carotene) intake is recommended.

In our case, vitamin A is expressed as retinol; nevertheless, it is seen that 100 g of spirulina grown in a bioreactor contains half of the daily intake of vitamin A. In the studied sample, vitamin B_2_ has a lower content compared to that reported by the authors of [35], approximately 3.5 mg g^−1^. Vitamin C is a water-soluble vitamin with antioxidant capacity which plays a significant role in tumor angiogenesis [36]. Vitamin C is not present in all samples of the mentioned cyanobacterium. Some studies indicate its presence, while others do not. The authors attribute this to different cultivation factors [37]. In the examined sample, this vitamin is present in an amount, approximately 10 mg/100 g.

**Scheme 2:**
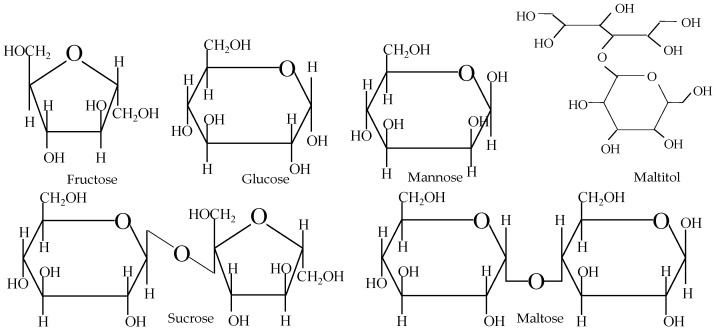
Carbohydrate structural formulas.

Carbohydrates can be found in the dry biomass of *Arthrospira platensis*. Their content in the sample is presented in Table 3. Their structural formulas are shown in Scheme 2.

Among all carbohydrates in the examined sample of *Arthrospira platensis*, the predominant monosaccharide is mannose, which is crucial for cellular communication and the immune response in living organisms. Following in content is maltitol, which can be effectively applied as a sweetener for people with diabetes. A significant portion of the carbohydrates extracted from the studied sample of spirulina can be used as ingredients in various pharmaceutical products (e.g., vitamins) or supplements, playing an important role in the biochemical processes in the body. The data obtained for Bulgarian spirulina grown in a bioreactor differ from those reported by an authors’ collective [22], which indicate approximately 14 times higher glucose content and a similarly lower mannose content. This fact could be due to the cultivation. 

**Scheme 3:**
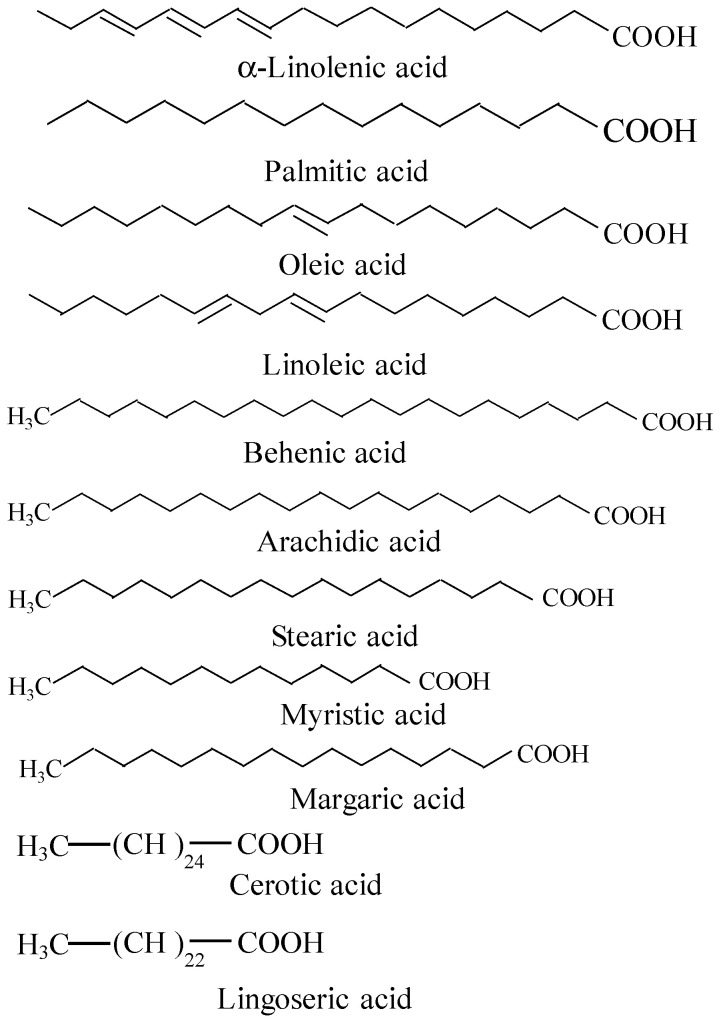
Structural formulas of fatty acids.

The following primary fatty acids were established from the identified lipids in one gram of dry mass, as presented in Table 4, and their structural formulas are shown in Scheme 3. Among the unsaturated fatty acids, the one with the highest content is α-linolenic acid (25.28%), while palmitic acid (22.55%) prevails among the saturated fatty acids. Arachidonic acid, the second-highest unsaturated fatty acid in terms of percentage, finds more applications in medicine and pharmacology than α-linolenic acid. It is integrated into non-steroidal anti-inflammatory drugs in formulations affecting vascular tone, blood coagulation, and others, exerting a favorable effect on the cardiovascular system. Its metabolites can influence the function of neuronal membranes and have a regulatory effect on neurotransmitters, which is crucial in treating certain psychiatric conditions. 

The ratio of n-6/n-3 is approximately 1. The most suitable ratio is between 1:1 and 1:4. The competition between n-6 and n-3 fatty acids during enzymatic action in the body is significant and often used to interpret biomarker data or to create dietary regimes [38]. Polyunsaturated fatty acids n-3 have a beneficial impact in treating cardiovascular diseases, diabetes, cancer, Alzheimer’s disease, dementia, and depression [39]. The naturalness of the studied sample can be highlighted, as the data show low stearic and behenic acid content, and gamma-linolenic acid was not detected [40]. High levels of linoleic acid in the blood plasma of women are presumed to correlate with a lower risk of breast cancer in women [41,42].

The chemical composition of *Spirulina* has been proven to depend on the geographic region, year, season, climatic conditions, environmental factors, cultivation, and processing methods, as well as the strain used in the bioreactor. The significant variability in research results regarding its chemical composition is also due to the diverse cultivation conditions (acidity, temperature, exposure to light, and drying methods) [43,44,45,46]. The chemical composition of 15 strains of *Spirulina*, cultivated in controlled environments across various countries (Ethiopia, Kenya, Chad, Mexico, USA, India, Peru, Spain), was investigated by Muhling et al. [43]. Similar to our study, the predominant fatty acids were palmitic and α-linolenic acids, together constituting 88–92% of the total fatty acids. The quantity of palmitic acid ranged from 42.3% to 47.6% among different strains, which is considerably higher than our findings of 22.55%. None of the *Spirulina* strains contained γ-linolenic acid. The authors observed variations in the percentage content of fatty acids depending on the strain of culture, cultivation location, temperature, light exposure, and other factors.

The fatty acid composition of a dry extract of *Spirulina* from a farm in Italy was analyzed, revealing a predominant content of five fatty acids: palmitic acid (64.1%), linoleic acid (13.7%), oleic acid (10.0%), γ-linolenic acid (6.2%), palmitoleic acid (4.1%), and stearic acid (1.9%). This content significantly differs from ours and can be attributed to the different conditions under which the *Spirulina* was cultivated. The protein content established by the authors, 54.84 g/100 g dw, is close to that found in our study, which was 50.16 g/100 g dw [45].

**Figure 3:**
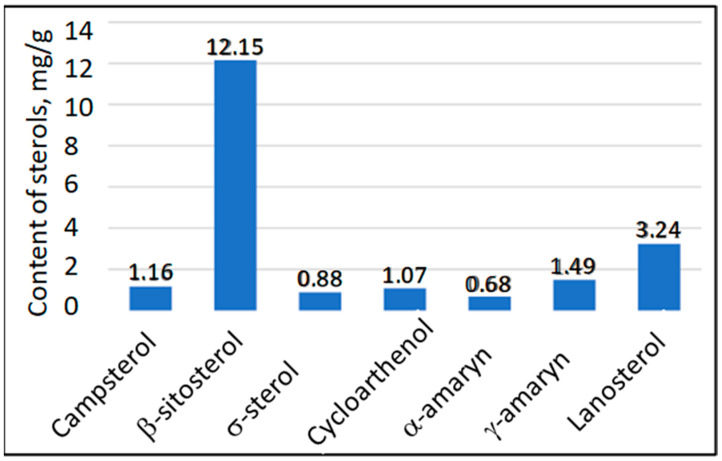
Sterol content in *Arthrospira platensis* grown in a bioreactor in Bulgaria.

In addition to fatty acids, the examined sample contains other lipids that are important biomolecules in inflammation-related cellular pathologies [47]. Sterols prevail, serving as a fundamental building unit in cellular membranes. Among the free sterols in the sample, β-sitosterol predominates (Figure 3). A similar observation has been noted in green algae such as *Ulva rigida* [48].

The concentrations of Mg, Fe, Mn, Cu, Ba, Ca, Cr, Sr, S, and Zn [22] in *Arthrospira platensis* produced in Bulgaria were determined. Calcium, S, and Mg are the main components with concentrations of 5.13 g kg^−1^, 4.83 g kg^−1^, and 2.57 g kg^−1^. The remaining elements follow the trend Fe > Zn > Mn > Cu ≈ Sr > Ba > Cr (317 > 242 > 55.0 > 25.4 ≈ 25.3 > 4.16 > 2.53 mg.kg^−1^); the RSD of the obtained results was 2–7%. Comparisons with the elemental compositions of other *Spirulina* are complicated since they are grown in different conditions—outdoors or indoors. Often, producers add additional soluble mineral compounds to the waters in which they are grown. Our results for S, Ca, Mn, Fe, and Sr completely fit within the range of the results of a large study of 46 *Spirulina* supplements purchased from various health food stores/supermarkets and online stores in Slovenia [41]. However, the Zn result exceeds their highest value of 52.7 mg kg^−1^, but other studies confirm the detection of high concentrations of Zn in spirulina—240–336 μg g^−1^ [49].

## 4. Conclusions

This study evaluated the metabolomic composition of *Arthrospira platensis*. The findings indicate that *Arthrospira platensis* cultivated in a Bulgarian reactor exhibits a high content of the following:

- Pyroglutamic and aspartic acid;
- Vitamins A and C;
- Mannose;
- α-linolenic acid, palmitic acid, and arachidonic acid;
- Calcium, sulfur, and magnesium.

In addition, various other organic molecules were also discovered. The analysis showed that the microcystins LR, RR, YR, and nodularin were below the 0.2 µg/L detection limit.

The knowledge of the metabolomic profile of *Arthrospira platensis* can serve for its incorporation into various types of foods to enhance their nutritional value, as well as for its inclusion in dietary supplements, either on its own or in combination with other plant species.

## Figures and Tables

**Table 1:** Main types of chemical substances in *Arthrospira platensis* grown in a bioreactor in Bulgaria.

	Our Data	Value from the Literature	Refs.
	Content ± SD, mg g^−1^ Dry Mass		
Phenolic acids			
Cinnamic acid	2.27 ± 0.34		
Salicylic acid	0.94 ± 0.14		
Carboxylic acids			
Pyroglutamic acid	9.51 ± 1.41		
Fumaric acid	0.69 ± 0.10		
Malic acid	2.41 ± 0.36		
Succinic acid	2.40 ± 0.35		
Phosphoritic acid	0.60 ± 0.09		
Non-essential amino acids			
Alanine	0.98 ± 0.05		
Asparatic acid	5.25 ± 0.78		
Essential amino acids			
Valine	0.82 ± 0.12	0.35	[21,22]
Leucine	0.78 ± 0.12	0.49	[21,22]
Isoleucine	0.57 ± 0.08	0.32	[21,22]
Methionine	0.64 ± 0.09	0.12	[21,22]
Threonine	0.62 ± 0.09	0.30	[21,22]
Conditional non-essential amino acids			
Norvaline	0.50 ± 0.07		
Proline	0.96 ± 0.14		
Serine	0.68 ± 0.10		

**Table 2:** Vitamin content in *Arthrospira platensis*.

Vitamin Content	Mean Value	Standard Deviation
Vitamin B_2_ (riboflavin), mg/100 g	1.01	0.25
Vitamin C (Ascorbic acid), mg/100 g	9.43	0.80
Vitamin E (α-tocopherol), mg/100 g	3.57	0.55
Vitamin A (retinol), μg/100 g	10.30	2.80

**Table 3:** Carbohydrate content of *Arthrospira platensis* grown in a bioreactor in Bulgaria.

Carbohydrate Content	Mean Value, mg g^−1^	Standard Deviation, mg g^−1^
Fructose	2.62	0.38
Glucose	3.61	0.54
Mannose	137.02	14.44
Sucrose	4.86	0.72
Maltose	50.61	7.78
Maltitol	88.87	14.42

**Table 4:** Fatty acids in *Arthrospira platensis* grown in a bioreactor in Bulgaria.

Fatty Acids	Mean Value mg g^−1^	Standard Deviation, mg g^−1^	Value %
α-Linolenic acid (C_18:3 n-3_)	15.59	2.44	25.28
Palmitic acid (C_16:0_)	13.91	3.07	22.55
Oleic acid (C_18:1_)	1.12	0.24	1.82
Linoleic acid (C_18:2 n-6_)	4.22	0.91	6.84
Cerotic acid	4.14	0.89	6.71
Lingoseric acid (C_24:0_)	2.83	0.61	4.59
Behenic acid (C_22:0_)	2.16	0.47	3.50
Arachidic acid (C_20:4 n-6_)	12.87	1.88	20.87
Stearic acid (C_18:0_)	3.11	0.67	5.04
Myristic acid (C_14:0_)	1.52	0.33	2.46
Margaric acid (C_17:0_)	0.22	0.05	0.37
∑SFA	27.88	-	45.21
∑USFA	33.80	-	54.79
∑n−3FA	15.59	-	25.28
∑n−6FA	17.09	-	27.71
n-6/n-3	1.10	-	1.10

## Data Availability

Datasets from the time of this study are available from the respective authors upon reasonable request.
